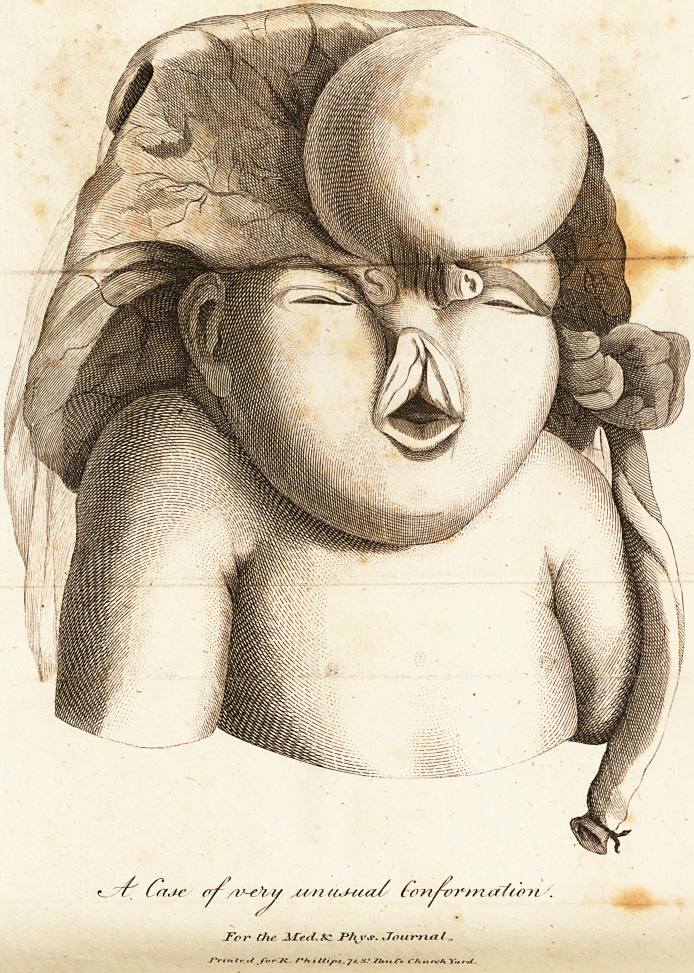# Case of Extraordinary Malformation in a Fætus

**Published:** 1800-05

**Authors:** T. Pole

**Affiliations:** Surgeon and Lecturer upon Midwifery, Leadenhall Street, London


### Medical and Physical Journal.

VOL. III.] MAY, 1800. [NO. XV.

### *Case of extraordinary Malformation in a Fœtus, with a Plate representing the same.*

*By* T. POLE, *Surgeon and Lecturer upon Midwifery, Leadenhall Street, London.*

ABOUT two or three months ago I received a large female fœtus, from some unknown friend in the country, without its being accompanied or followed by any letter which could inform me to whom I am indebted for so curious and acceptable an addition to my Museum. I indulge myself with a hope that this publication may meet the eye of the person who has generously presented me with it: and, should that be the case, I shall be further obliged by his transmitting me some account of the presentation and delivery; also, whether the child was born alive; and, if so, how long it lived; with any other circumstances respecting it, which may have occurred to his observation; and such information may serve as an appendix to the present description of the phænomenon, which I have no doubt will be acceptable to the readers of this valuable and instructive Journal.

The case which I now communicate to the public is, in some respects, not singular; but in some others, as far as I have seen, is entirely so.—The bones of the head, above the orbits, appear almost entirely deficient; there were some small portions of the ossa frontis loose and moveable above the globes of the eyes, which latter were concealed by the pressure upon the eyelids: the cutis, on the right side, over the temple and ear, was pressed outward in the form of a tumour. The mouth was stretched open, by the centre of the upper lip being drawn upward to a great extent, which exposed the upper gums; these, as well as the lip, formed a sort of angle in that part of the face where the nose is usually situated: There was in fact no nose, but the two nostrils opened near the internal angles of the eyes on each side; that on the left side appeared in the extremity of a projection, somewhat resembling a proboscis; that on the right, formed a longer and more incurvated orifice, with its edges in fome degree projecting from an irregular furface. From this noftril was continued to the angle of the upper lip, a prominent ridge, conftituted by a ftretching of the cutis, rather than any bony fubftance refembling the offa nafi. The fummit of the cranium being entirely deficient, there was not fufficient fpace for containing the whole of the brain. This want of room was fupplied by a large fpherical facculus covered by the common cutis, fituated principally over the left eye, and occupying the place of the left os frontis. It was about the fize of a goofe's egg, conftituting an hernia cerebri. This tumour was very foft, falling in whatever direction the head was placed, and was connected by a bafe of about an inch and a half in diameter. The occiput was tolerably well formed, but appeared to be deficient towards its upper edge. The neck of the child was remarkably fhort, and the thorax very prominent anteriorly.

The moft fingular circumftance in this cafe, is the attachment of the placenta to the upper part of the child's head, (diftinguifhed in the plate by a dark line paffing from the left noftril to the top of the ear on the fame fide) which was not by mere membranous union, but of its more folid and compact parts. The largeft portion of the placenta lay over the occiput and fcapulæ, and extended confiderably to the right and left fide of the head, fo that in viewing the child, as I have reprefented it in the drawing, its internal furface only is feen, with the upper edge bent backward: this attachment of the placenta was principally toward the right fide of the head. The umbilical chord came from the placenta on the left fide, and appeared to take its courfe down the fame fide of the body to the umbilicus, from which it had been feparated before I received it; about fix or eight inches of it were left to the placenta when it came to my hands: near to the extremity of the chord a ligature was applied. At a fmall diftance from the origin of the funis there was a remarkable convolution of the umbilical veffels, which give it a peculiar appearance, as in the plate.

From the placenta and funis umbilicalis having undergone fome degree of putrefaction, and their appearing confiderably drier than might have been expected, and from the child being evidently free from any putrefactive change, I have been induced to conclude, that it was not only born alive and healthy, but that it had lived fome days; which I regret not having it in my power at prefent to fpeak more fully upon, as I have not yet been favoured with any particulars by the perfon who delivered the mother, and was witnefs to all the circumftances refpecting the child.

Children

Children born without the upper part of the cranium, conſtitute no uncommon ſpecies of monſter, ſeveral ſpecimens of which I have now in my collection; I have alſo ſeen one inſtance of an attachment of the placenta to the abdomen, by an extenſive ſurface, having a funis which appeared to paſs to the umbilicus, a termination which was concealed by the attachment of the placenta: But I had not ſeen or heard of an attachment to the head, until the preſent caſe came under my notice. Nature is, however, ſo frequently regardleſs of her common laws, as to deviate in ſuch an endleſs variety, that the beſt informed man can have no adequate idea of the diverſity of her productions.

## Figures and Tables

**Figure f1:**